# Are people most in need utilising health facilities in post-conflict settings? A cross-sectional study from South Kivu, eastern DR Congo

**DOI:** 10.1080/16549716.2020.1740419

**Published:** 2020-03-19

**Authors:** Espoir Bwenge Malembaka, Hermès Karemere, Ghislain Bisimwa Balaluka, Chiara Altare, Magdalene Akos Odikro, Samuel Makali Lwamushi, Rosine Bigirinama Nshobole, Jean Macq

**Affiliations:** a Ecole Régionale de Santé Publique, ERSP, Faculté de Médecine, Université Catholique de Bukavu, Bukavu, Democratic Republic of Congo; b Institute of Health and Society, IRSS, Université Catholique de Louvain, Brussels, Belgium; c Centre for Humanitarian Health, Department of International Health, Johns Hopkins Bloomberg School of Public Health, Baltimore, MD, USA; d Ghana Field Epidemiology and Laboratory Training Programme (GFELTP), Department of Epidemiology and Disease Control, University of Ghana, Accra, Ghana

**Keywords:** Health facility, utilisation, health need, post-conflict, South Kivu, DR Congo, WHODAS

## Abstract

**Background**: The disruptive effect of protracted socio-political instability and conflict on the health systems is likely to exacerbate inequities in health service utilisation in conflict-recovering contexts.

**Objective**: To examine whether the level of healthcare need is associated with health facility utilisation in post-conflict settings.

**Methods**: We conducted a cross-sectional study among adults with diabetes, hypertension, mothers of infants with acute malnutrition, informal caregivers (of participants with diabetes and hypertension) and helpers of mothers of children acutely malnourished, and randomly selected neighbours in South Kivu province, eastern DR Congo. Healthcare need levels were derived from a combination, summary and categorisation of the World Health Organisation Disability Assessment Schedule 2.0. Health facility utilisation was defined as having utilised in the first resort a health post, a health centre or a hospital as opposed to self-medication, traditional herbs or prayer homes during illness in the past 30 days. We used mixed-effects Poisson regression models with robust variance to identify the factors associated with health facility utilisation.

**Results**: Overall, 82% (n = 413) of the participants (N = 504) utilised modern health facilities. Health facility utilisation likelihood was higher by 27% [adjusted prevalence ratio (aPR): 1.27; 95% CI: 1.13–1.43; *p* < 0.001] and 18% (aPR: 1.18; 95% CI: 1.06–1.30; *p* = 0.002) among participants with middle and higher health needs, respectively, compared to those with low healthcare needs. Using the lowest health need cluster as a reference, participants in the middle healthcare need cluster tended to have a higher hospital utilisation level.

**Conclusion**: Greater reported healthcare need was significantly associated with health facility utilisation. Primary healthcare facilities were the first resort for a vast majority of respondents. Improving the availability and quality of health service packages at the primary healthcare level is necessary to ensure the universal health coverage goal advocating quality health for all can be achieved in post-conflict settings.

## Background

The sustainable development goals (SDG) target 3.8 advocates the principle of universal health coverage (UHC), stressing the importance of health services provision to all people in need in a way that ensures the health and wellbeing of individuals and communities are improved at an affordable cost [1]. Inequitable utilisation of health facilities continues to pose a major threat to the progress towards UHC in resource-constrained settings, particularly in post-conflict contexts [2,3]. Reports from high and low middle-income countries (LMIC) suggest higher utilisation of healthcare services by people with higher socio-economic status [4–6] (vertical inequality). Although modern health facility utilisation (HFU) is not an end on its own, it indicates that people are seeking care and accessing facilities where services are likely to be available and of acceptable quality.

Globally, there is growing recognition of the need for health systems to shift from being disease-centred to assuming a broader and person-centred perspective that accounts for social and environmental factors, as well as for the uniqueness and complexity of each person’s life, needs and goals [7–11]. However, the definition of essential health services and the notion of equity in access to healthcare are not yet person-centred in many LMICs [12,13], where disease control and mortality reduction for specific population groups are still at the centre. A recent study conducted in the Democratic Republic of Congo (DRC) province of South Kivu premised on a broader view of health and proposed a new way of defining health status and care needs at community level in LMIC by focussing on people’s capacities and abilities to cope with day-to-day physical, social and emotional challenges [14]. By approaching individual health through measurement of performance in various life domains, including cognition, mobility, self-care, getting along with others, domestic and professional duties, social life and participation, the study revealed a clear needs gradient among community members which goes beyond the mere presence or absence of disease or disability.

Ensuring equitable utilisation of healthcare according to needs remains a challenge in post-conflict, resource-constraint settings like the DRC. The health system has been fragilised by nearly three decades of socio-political turmoil in the eastern provinces. Because of decreasing public funding and insufficient leadership, health financing heavily relies on out-of-pocket payments and external aid (mainly humanitarian assistance) [15]. The distribution of health service utilisation is likely to be skewed towards the richest in urban areas [15], potentially perpetuating the inverse care law pattern [16,17] whereby the health service utilisation balance is deteriorated against those most in need, leaving individuals in complex medico-psychosocial situations behind.

In the era of the SDG 3.8, assessing the link between health needs and health facility utilisation is necessary for the development of health systems that are responsive to people’s need for autonomy, self-management and coping with life’s physical, emotional, and social challenges [18]. This is even more true in resource-limited and post-conflict settings where horizontal inequalities can also contribute to instability. Given that protracted socio-political instability and conflict are likely to exacerbate inequities in health service utilisation in post-conflict and fragile contexts, in this study we set to examine the association between healthcare needs and modern health facility utilisation (HFU) in South Kivu.

## Methods

### Study design, settings and population

This cross-sectional study was carried out in four health zones (HZ) of South Kivu Province in eastern DRC: one semi-urban (Bagira) and three rural (Miti-Murhesa, Katana and Walungu). Bagira HZ is located on the periphery of the province capital (Bukavu). Katana and Miti-Murhesa HZ span the national road connecting Bukavu and Goma, the two major cities of the Kivu. Walungu was one of the most conflict-affected HZ in the previous decades. The instability in the Kivu exacerbated population poverty, caused the collapse of local markets and diminished livestock production to almost its disappearance [19], thus creating the conditions for a vicious cycle of socio-political instability [20]. From the health system point of view, each province in DRC is organized in health zones, and these in turn in health areas (HA). Within each HZ primary healthcare services are offered through health centres providing the minimum service package. This focuses on maternal and child preventive and curative care [15]. There are on average 15 HA per health zone in South Kivu. Secondary care is provided at HZ level hospitals offering inpatient and reference services, in addition to technical support to health centres through integrated supervision.

Six HA (estimated population of 105,047 inhabitants in 2017) were purposively selected for this study based on security constraints, geographical accessibility and availability and quality of data at the health centres. The study population is comprised of three separate groups: 1) the tracer group: i.e. adults in one of the following conditions: either being a mother of a child with global acute malnutrition (defined as MUAC ≤11.5 or nutritional oedema); or an individual with history of self-reported diabetes and/or hypertension; 2) their caregiver (or helper of mothers with a malnourished child), and 3) a randomly selected neighbour. Participants were aged at least 15 years. These sub-population categories were defined to gain better insight into different perspectives of health disability levels observable at primary healthcare facilities in eastern DRC. The likelihood of complex health situations spanning the medical, psychological and social spheres of an individual’s life was deemed high in these sub-populations [21,22]. The selected health conditions are prevailing in South Kivu [23–25], are relatively easy to identify and manageable even at primary care level when without complications.

### Sampling procedure and selection of participants

This study is nested in a broader research project that aims to investigate to what extent modifying how health services are provided at primary care level can influence the perceived health status of community members. For the current analysis, we used data from the baseline cross-sectional survey of the cohort that is enrolled in the main research project. The baseline study was conducted in villages around six health centres in the selected health areas. Within villages, households with individuals with self-reported diabetes, hypertension or child acute malnutrition were pre-identified by community health workers. Subsequently, one caregiver living in the same household as the participant with the tracer condition and one random neighbour were selected by trained data collectors (locally recruited nurses). In each village, data collectors were guided by community health workers during the door-to-door visits. Further details on this methodology are provided elsewhere [14].

Overall, 1266 participants who provided valid data on cognitive, functional and social disability were included in the main research study. Of these, we extracted a sub-sample of 553 participants who reported an acute illness during the 30 days preceding the interview for the current study. A set of questions were asked about the history of a possible acute illness in the past 30 days prior to the interview and if and where treatment was sought in the first resort. Questions covered a broad range of acute health conditions, either self-diagnosed or diagnosed by a health professional, such as fever, diarrheal, abdominal or generalized pain, difficulty in breathing, pneumonia, syncope, malaria, flu-like syndrome or any other acute conditions (to be specified if possible). We used this inclusion criteria to reduce recall bias.

### Data collection and variables

A structured and pre-tested questionnaire was used to collect data on socio-demographics, social integration and health facility utilisation between December 2017 and March 2018. The 36-item WHODAS 2.0 questionnaire was used to examine the cognitive, functional and social disability of the participants [26]. WHODAS 2.0 is a standard tool with cross-cultural validity extensively used across WHO regions to measure the impact of any health condition in terms of performance in a given life domain. WHODAS 2.0 covers following domains: cognition (understanding and communicating), mobility (moving and getting around), self-care (ability to attend to personal hygiene and safety), getting along (interacting with other people), life activities (home responsibilities, leisure, work and school) and social participation (joining in community activities, social participation and engagement). The WHODAS 2.0 was translated to and back translated from Kiswahili (the national language widely spoken in eastern DRC), to ensure cross-cultural and conceptual equivalence [27]. Details about the translation process are provided elsewhere [14]

WHODAS 2.0 measures decrement in performance in life domains making it a valuable instrument for health needs identification and monitoring; interventions design, and assessment of their outcomes and effectiveness; priority setting and resource allocation [26]. WHODAS 2.0 was used to define the main predictor variable under this study. A principal component analysis of the seven summary scores of the WHODAS 2.0 domains was performed with the use of the FactoMineR software package in R, prior to a hierarchical clustering of the principal components and guided by Ward’s method [28]. Three health needs clusters were generated and used as a three-level ordinal variable reflecting the healthcare needs gradient corresponding to low, moderate and high disability levels.

#### Dependent variable

The primary outcome is modern health facility utilisation, a binary variable referring to whether or not a person has utilised in the first resort a health post, a health centre or a hospital as opposed to self-medication, traditional herbs or prayer homes during the illness episode in the 30 days preceding the survey.

As secondary outcomes, two additional binary variables were defined as follows: 1) utilisation of primary health facilities (coded as 1 if a person reported having utilised as a first resort either a health post or centre during the 30 days prior to the interview, and as 0 if otherwise); 2) hospital utilisation coded as 1 if a person reported having utilised as a first resort a secondary level health facility during the 30 days prior to the interview, and as 0 if otherwise. Primary health facilities are officially run by nurses and offer the minimum package of activity. Secondary level health facilities are those, both public and private, with permanently appointed medical doctors, offering the complementary package of activity through hospitalisation and reference services.

#### Independent variables

A wealth status variable was created in three steps. First, a Multiple Correspondence Analysis was run on housing characteristics (pavement and permanent, semi-permanent or temporary structure) and ownership of a television, a radio, a computer, a manufactured bed, small animals, cattle, land, a bicycle, a motorcycle to create a socio-economic index. Five socio-economic quintiles were then derived from wealth indices, following an approach commonly used in Demographic and Health Surveys conducted in low- and middle-income countries. To create the three socio-economic classes (least poor, middle poor, poorest), the two lowest (poorest 40%) and the two middle (40%) quintiles were, respectively, merged as suggested by Filmer and Pritchett [29].

Social cohesion and networking have been shown to be important social determinants of health and primary healthcare utilisation [30,31]; they were approximated in this study by belonging to local social and saving cooperatives and attendance to religious activities.

Other independent variables included in the analyses were sociodemographic characteristics (sex and age of the respondent, occupation of the head of household), health zone of residence and the enrolment status of the participant (as described above, either an adult with self-reported chronic morbidity or a mother of child with acute malnutrition, a caregiver and a randomly selected neighbour).

### Statistical analyses

Chi-squared and Student’s t tests were used to compare the general characteristics of the study population by HFU. To examine the association between HFU and health needs clustering, we run bivariable and multivariable mixed-effects Poisson regression models with robust variance. In these models, health needs level was treated as a fixed effect and health area as a random effect to account for the intrinsic non-independence of observations in the same health area. The choice of a modified Poisson regression over a logistic regression was justified by the fact that in presence of common outcomes (i.e. prevalence higher than 10%), odds ratios derived from logistic regression tend to bias the association away from the null. In such a scenario, alternative models directly estimating prevalence ratio (PR) are preferred to generate more accurate point and interval estimates [32,33]. All the study variables examined in the univariate regression models were maintained in multivariable models based on either a conservative p-value ≤0.2 or on public health plausibility. Multicollinearity between explanatory variables was suspected on the basis of a variance inflation factor greater than 10. We used Stata 15 for the analyses. The significance was set to p < 0.05 (two-sided test).

### Ethical considerations

Ethical clearance was obtained from the Faculty of Medicine of Université Catholique de Bukavu and the Université Catholique de Louvain. Written informed consent was received from all participants, either by signature or fingerprint, depending on literacy. Child assent was obtained for respondents below 18 years of age, after a parent or guardian’s consent. The research tools, including the consent forms, were translated to Kiswahili. Data collectors were trained on ethics and were requested to fully read out and explain the aim of the study to the respondents prior to enrolment in the study. Participants were clearly informed they were free to drop out of the interview at any time they would deem it necessary.

## Results

### Characteristics of the study participants

It was found that 59.5% of the study participants were enrolled in the tracer group; neighbours and informal caregivers were 20.9% and 19.6%, respectively. Over half of the participants (52.2%) were in the lower health need level and 18.9% in the highest health needs level. The mean age was 53.2 (SD: 18.2), men were more represented in the study sample (67.1%) and 56.1% of the participants reported attending church at least twice a week. HFU was dependent on the health needs level (p < 0.001) and unrelated to wealth class (*p* = 0.772) (Table 1).

**Table 1.** Characteristics of the study participants by health facility utilisation status

	Health facility utilisation			
Characteristics	Yes	No	Total	P value
Enrolment status (N = 445)				0.195
Neighbours	78 (83.9)	15 (16.1)	93 (20.9)	
Informal caregivers	66 (75.9)	21 (24.1)	87 (19.6)	
Tracer group	223 (84.2)	42 (15.8)	265 (59.5)	
Healthcare needs and disability level (N = 418)				<0.001
Low	159 (72.9)	59 (27.1)	218 (52.2)	
Moderate	111 (91.7)	10 (8.3)	121 (29.0)	
High	63 (79.8)	16 (22.2)	79 (18.9)	
Zone (N = 519)				<0.001
Bagira	127 (79.9)	32 (20.1)	159 (32.5)	
Miti-Murhesa and Katana*	172 (76.4)	53 (23.6)	255 (46.0)	
Walungu	99 (94.3)	6 (5.7)	105 (21.5)	
Sex (N = 499)				0.445
Female	131 (79.9)	33 (20.1)	164 (32.9)	
Male	277 (82.7)	58 (17.3)	335 (67.1)	
Age (mean), (N = 494)	54.8 (17.7)	52.1 (18.8)	53.2 (18.2)	0.195
Age group (N = 494)				0.124
<40 years	89 (76.1)	28 (23.9)	117 (23.7)	
40–59 years	132 (85.7)	22 (14.3)	154 (31.2)	
≥60 years	183 (82.1)	40 (17.9)	223 (54.1)	
Saving membership (N = 504)				0.277
Yes	315 (80.8)	75 (19.2)	390 (78.2)	
No	93 (85.3)	16 (14.7)	109 (21.8)	
Weekly church attendance (N = 483)				0.011
≤1 time/week	163 (76.9)	49 (23.1)	212 (43.9)	
2–3 times	135 (83.9)	26 (16.1)	161 (33.3)	
≥4 times	99 (90)	11 (10)	110 (22.8)	
Chronic morbidity (N = 467)				0.021
Absent	139 (76.0)	44 (24.0)	183 (39.1)	
Diabetes or hypertension	214 (84.9)	38 (15.1)	252 (53.9)	
Diabetes and hypertension	30 (90.9)	3 (9.1)	33 (7.0)	
Wealth class (N = 504)				0.772
Least poor	86 (83.3)	16 (15.7)	102 (20.2)	
Middle poor	175 (81)	41 (19)	216 (42.9)	
Poorest	152 (81.7)	34 (18.3)	186 (36.9)	
Head of household’s occupation (N = 459)				0.706
Formal work	54 (87.1)	8 (12.9)	62 (13.5)	
Informal work	67 (81.7)	15 (18.3)	82 (17.9)	
Petty trade/farming	175 (80.7)	42 (19.3)	217 (47.3)	
No occupation	81 (82.7)	17 (17.3	98 (21.3)	

Data are n (%), unless otherwise specified. *: Miti-Murhesa and Katana health zones are grouped since they were formerly constituting the Katana health zone. They share the same characteristics in terms of health coverage, location and level of past exposure to conflict. Enrolment of the participants spanned villages across the border between the two health zone borders.

### Factors associated with health facility utilisation

Among the study participants who reported being sick 30 days prior to being interviewed, 18.1% did not use any formal health facility, rather resorted to traditional healers, prayer homes or self-medication; 12.1% went to hospitals (Table 2).

**Table 2.** Health facility utilisation among study participants who reported being sick 30 days prior to the survey

Health facility type	N (%)
Health post	36 (7.1)
Public health centre in the health area	248 (49.2)
Private health centre	68 (13.5)
Hospital	61 (12.1)
Traditional healers	16 (3.2)
Prayer homes	5 (1.0)
Self-medication and other	70 (13.9)

Health needs level was significantly associated with high HFU. In fact, the level of HFU was higher by 27% [adjusted prevalence ratio (aPR): 1.27; 95% CI: 1.13–1.43; *p* < 0.001] and 18% (aPR: 1.18; 95% CI: 1.06–1.30; *p* = 0.002) among participants with middle and higher health needs, respectively, compared to those with lower health needs (Table 3). Other factors independently and positively associated with HFU were attending church services at least four times a week compared to attending once or less (aPR: 1.17; 95% CI: 1.15–1.20; *p* < 0.001), and being in the middle wealth group compared to being in the poorest group (aPR: 1.12; 95% CI: 1.06–1.19; *p < *0.001). Factors independently and negatively associated with HFU were the health status of the participant at enrolment, with caregivers (aPR: 0.77; 95% CI: 0.68–0.88; p < 0.001) and participants in the tracer group (aPR: 0.73; 95% CI: 0.57–0.95; p = 0.017) being less likely to utilise health facilities than neighbours; and younger age (aPR: 0.997; 95% CI: 0.995–0.999; p = 0.017).

Analysis of secondary outcomes is reported in Tables 4 and 5. Compared to those with lower health needs, participants with middle health needs were less likely to use primary health facilities (PR: 0.93; 95% CI: 0.88–0.99; *p* = 0.015) and much more likely to use hospitals (PR: 2.17; 95% CI: 1.44–3.29; *p < *0.001). Additionally, participants in the tracer group (PR: 3.75; 95% CI: 1.22–11.57; *p* = 0.021) and their caregivers (PR: 6.55; 95% CI: 5.25–8.18; *p* < 0.001) had significantly higher hospital utilisation levels compared to neighbours.

**Table 3.** Factors associated with health facility utilisation

Variable	Unadjusted PR (95% CI)	P value	Adjusted PR (95% CI)	P value
Healthcare needs and disability level				
Low	Ref.		Ref.	
Moderate	1.27 (1.10–1.48)	0.002	1.27 (1.13–1.43)	<0.001
High	1.11 (1.01–1.20)	0.022	1.18 (1.06–1.30)	0.002
Enrolment status				
Neighbours	Ref.		Ref.	
Caregivers	0.90 (0.86–0.95)	<0.001	0.77 (0.68–0.88)	<0.001
Tracer group	1.00 (0.91–1.11)	0.939	0.73 (0.57–0.95)	0.017
Health zone				
Bagira	Ref.		Ref.	
Miti-Murhesa and katana	0.96 (0.92–0.99)	0.025	0.92 (0.74–1.14)	0.462
Walungu	1.18 (1.13–1.23)	<0.001	1.18 (0.90–1.53)	0.233
Education (years)	1.00 (0.99–1.02)	0.657	0.99 (0.97–1.02)	0.688
Sex				
Male	Ref.		Ref.	
Female	1.04 (0.98–1.11)	0.206	1.06 (0.96–1.17)	0.238
Age	1.00 (1.00–1.00)	0.162	1.00 (0.99–1.00)	0.017
Saving membership				
No	Ref.		Ref.	
Yes	1.05 (0.97–1.15)	0.222	0.98 (0.88–1.10)	0.773
Church attendance				
≤1 time/week	Ref.		Ref.	
2–3 times	1.10 (1.03–1.17)	0.003	0.98 (0.90–1.06)	0.616
≥4 times	1.19 (1.02–1.39)	0.029	1.17 (1.15–1.20)	<0.001
Occupation of head of household				
Formal salaried	Ref.		Ref.	
Informal worker	0.94 (0.82–1.08)	0.356	1 (0.71–1.40)	0.985
Petty trade/farming	0.92(0.83–1.02)	0.129	0.89 (0.70–1.14)	0.362
No occupation	0.95 (0.87–1.05)	0.302	0.87 (0.64–1.18)	0.375
Wealth class				
Poorest	Ref.		Ref.	
Middle poor	1.00 (0.89–1.11)	0.940	1.12 (1.06–1.19)	<0.001
Least poor	1.03 (0.87–1.22)	0.702	1.06 (0.87–1.31)	0.548
Chronic morbidity				
Absent	Ref.		Ref.	
Diabetes or hypertension	1.12 (1.05–1.19)	<0.001	1.14 (0.92–1.41)	0.234
Diabetes and hypertension	1.20 (1.09–1.32)	<0.001	1.24 (0.95–1.62)	0.114

Ref.: reference category. The factors included in the multivariable regression analysis are healthcare needs and disability level, enrolment status, health zone of residence, education (years of schooling), being member of a local saving organisation, church attendance, occupation of the head of household, wealth class and history of chronic morbidity (diabetes and hypertension).

**Table 4.** Factors associated with the utilisation of primary healthcare facilities

Variable	Unadjusted PR (95% CI)	P value	Adjusted PR (95% CI)	p value
Healthcare needs and disability level				
Low	Ref.		Ref.	
Moderate	0.93 (0.85–1.03)	0.154	0.93 (0.88–0.99)	0.015
High	0.84 (0.71–1.00)	0.050	1.00 (0.83–1.19)	0.970
Enrolment status				
Neighbours	Ref.		Ref.	
Caregivers	0.89 (0.80–0.99)	0.026	0.85 (0.76–0.94)	0.003
Tracer group	0.88 (0.75–1.04)	0.129	0.93 (0.80–1.07)	0.290
Health zone				
Bagira	Ref.		Ref.	
Miti-Murhesa and katana	1.01 (0.92–1.11)	0.813	0.78 (0.56–1.08)	0.133
Walungu	1.20 (1.14–1.26)	<0.001	1.01 (0.80–1.28)	0.915
Education (years)	0.99 (0.97–1.00)	0.13	1.00 (0.98–1.02)	0.927
Sex				
Male	Ref.		Ref.	
Female	0.96 (0.90–1.02)	0.182	1.03 (0.94–1.11)	0.562
Age	1.00 (1.00–1.00)	0.943	1.00 (1.00–1.00)	0.620
Saving membership				
No	Ref.		Ref.	
Yes	0.97 (0.88–1.06)	0.45	0.91 (0.85–0.98)	0.013
Church attendance				
≤1 time/week	Ref.		Ref.	
2–3 times	1.03 (0.93–1.14)	0.553	1.25 (1.18–1.32)	<0.001
≥4 times	0.99 (0.94–1.05)	0.851	1.11 (1.03–1.18)	0.004
Occupation of head of household				
Formal salaried	Ref.		Ref.	
Informal worker	1.27 (1.08–1.49)	0.004	1.13 (0.92–1.39)	0.254
Petty trade/farming	1.32 (1.03–1.70)	0.028	1.32 (1.15–1.52)	<0.001
No occupation	1.16 (0.99–1.35)	0.069	1.02 (0.75–1.39)	0.883
Wealth class				
Poorest	Ref.		Ref.	
Middle	0.99 (0.95–1.02)	0.407	1.09 (0.98–1.21)	0.094
Least poor	0.84 (0.71–0.98)	0.028	1.00 (0.86–1.17)	0.977
Morbidity				
Absent	Ref.		Ref.	
Diabetes or hypertension	0.93 (0.79–1.08)	0.348	0.79 (0.69–0.91)	0.001
Diabetes and hypertension	0.80 (0.64–1.00)	0.047	0.76 (0.57–1.01)	0.06

Ref.: reference category. Primary health facilities include health posts and health centres. The factors included in the multivariable regression analysis are healthcare needs and disability level, enrolment status, health zone of residence, education (years of schooling), being member of a local saving organisation, church attendance, occupation of the head of household, wealth class and history of chronic morbidity (diabetes and hypertension).

**Table 5.** Univariable and multiple regression analysis of the association between health need level and hospital utilisation

Variable	Unadjusted PR (95% CI)	P value	Adjusted PR (95% CI)	P value
Healthcare needs and disability level				
Low	Ref.		Ref.	
Moderate	2.38 (1.53–3.72)	<0.001	2.17 (1.44–3.29)	<0.001
High	2.92 (2.43–3.51)	<0.001	1.30 (0.87–1.93)	0.194
Enrolment status				
Neighbours	Ref.		Ref.	
Caregivers	2.25 (0.92–5.49)	0.076	6.55 (5.25–8.18)	<0.001
Tracer group	3.72 (1.53–9.07)	0.004	3.75 (1.22–11.57)	0.021
Health zone				
Bagira	Ref.		Ref.	
Miti-Murhesa and katana	0.91 (0.60–1.40)	0.674	3.69 (0.42–32.85)	0.241
Walungu	0.19 (0.04–1.096)	0.044	1.04 (0.24–4.42)	0.962
Education (years)	1.07 (0.98–1.17)	0.149	0.99 (0.88–1.11)	0.849
Sex				
Male	Ref.		Ref.	
Female	1.28 (0.87–1.90)	0.209	0.98 (0.45–2.10)	0.952
Age	1.01 (1.00–1.02)	0.163	1.0 0 (0.98–1.02)	0.944
Saving membership				
No	Ref.		Ref.	
Yes	1.27 (0.89–1.83)	0.193	1.38 (0.59–3.22)	0.454
Church attendance				
≤1 time/week	Ref.		Ref.	
2–3 times	0.99 (0.50–1.96)	0.979	0.21 (0.07–0.63)	0.005
≥4 times	1.15 (0.74–1.79)	0.535	0.70 (0.55–0.89)	0.004
Occupation of head of household				
Formal salaried	Ref.		Ref.	
Informal worker	0.46 (0.28–0.75)	0.002	0.89 (0.08–9.40)	0.919
Petty trade/farming	0.29 (0.11–0.76)	0.011	0.27 (0.12–0.61)	0.001
No occupation	0.78 (0.53–1.13)	0.188	1.06 (0.10–10.83)	0.96
Wealth class				
Poorest	Ref.		Ref.	
Middle	0.98 (0.72–1.33)	0.887	0.66 (0.22–1.95)	0.450
Least poor	2.23 (1.21–4.10)	0.010	1.00 (0.33–3.05)	0.995
Morbidity				
Absent	Ref.		Ref.	
Diabetes or hypertension	2.61 (1.16–5.87)	0.021	3.64 (1.82–7.27)	<0.001
Diabetes and hypertension	4.72 (1.77–12.58)	0.002	4.77 (1.52–14.95)	0.007

Ref.: Reference category. The factors included in the multivariable regression analysis are healthcare needs and disability level, enrolment status, health zone of residence, education (years of schooling), being member of a local saving organisation, church attendance, occupation of the head of household, wealth class and history of chronic morbidity (diabetes and hypertension).

## Discussion

This community-based cross-sectional study is one of the first studies to apply a comprehensive approach to measuring healthcare needs and its association with health facility utilisation at community level in post-conflict settings. We investigated HFU in a heterogeneous group composed of adults with diabetes, hypertension, mothers of children with acute malnutrition, their informal caregivers and randomly selected neighbours. The majority of them utilized health facilities as a first resort in case of illness. Informal caregivers and participants in the tracer group were less likely to utilise modern health facilities compared to neighbours. Participants with moderate and higher level of health needs and disability were also more likely to utilise modern health facilities compared to those in with lower health needs and disability level. They tended to seek care at hospital level rather than at primary healthcare facilities, possibly expecting a more comprehensive management of their condition.

The level of HFU observed in this study is slightly higher than the 76.7% reported in a Kenyan study [34]. This relatively high HFU could be an effect of a selective sample of people with known morbidities. However, data from a study conducted in another eastern DRC province showed a similarly high HFU [35], suggesting additional explanations are plausible. For example, in post-conflict settings, a considerable number of humanitarian actors provide financial and capacity building support to health facilities, thus helping them remain functional. Despite (or thanks to) the ongoing conflict, the health system in South Kivu is the best funded in the country, if humanitarian aid is taken into account [15]. Other factors such as population resilience and adaptation capacity of health providers might also contribute to maintain this level of HFU in health zones of South Kivu that are progressively recovering from conflict. Further research to inform health service provision strategies in post-conflict settings is needed for cross-country learning.

Participants enrolled in the tracer group and their caregivers were unexpectedly less likely to utilise modern health facilities, but had higher hospital utilisation level than their neighbours. This may reflect the traditional organisation of healthcare services whereby chronic diseases are managed at the hospital level in most LMIC settings [36,37], including South Kivu. However, most vertical or selective donor-funded health programs are disproportionately focused on mortality reduction and disease indicators rather than on person-centred approaches of healthcare service organisation. This may lead to a shrinking role of primary healthcare for chronic patients and an increased utilisation of costly hospital-based curative services in post-conflict settings where the government leadership and health regulatory powers are weak. Further research is needed to understand how primary healthcare priorities for chronic conditions should be set in conflict-affected settings to overcome the potential competing visions between international donors’ vertical approaches and local government ambitions of building strong and sustainable primary healthcare systems [38–40].

Being part of a social network can influence health-seeking behaviour and healthcare service utilization. All studied health centres facilitate social initiatives to enhance the economic and social capital of vulnerable community members. These include, for example, the small-scale village saving and loan cooperative AVEC (from French ‘Association Villageoise d’Epargne et de Credit’) or social support clubs for the elderly. Although these initiatives make the health centre pivotal to community dynamics and are expected to encourage their members to resort to modern health facility services [41,42], we could not find such an effect in our study. The lower HFU observed among members of local saving cooperatives could reflect a reverse causation whereby those joining saving cooperatives are more likely to be young and economically active, thus less prone to severe health problems that would require health care. It could also mirror the positive effects of social integration and connectedness on self-esteem, sense of well-being, social competence, self-efficacy in management of chronic conditions, depression and stress responses [30].

Questions about the link between religion and health have often raised stormy debates [43]. Attendance of religious services has been associated with modern HFU and positive health outcomes [44–46]. Our finding may reflect high religious service attendance among participants with higher perceived health needs and vulnerability. In South Kivu, many health facilities are owned by and located near churches. Religious services attendance may also be an opportunity for social interaction, integration, and positive attitudes sharing, which can, in turn, exert a positive influence on health-seeking behaviours [44,47,48].

The association between wealth status and modern HFU [4,49] is not clear in our study. While respondents in the middle wealth level seemed to have higher utilization than those in the poorest group, utilization of primary health care and hospitals was comparable across wealth levels, as it was also the case in a study from Kenya [34]. This may be due to little heterogeneity across the asset-based wealth index in this predominantly rural population. Furthermore, in some post-conflict health zones of the Kivu, specific health services related to chronic diseases are subsidised by humanitarian organisations and are provided free of charge irrespectively of the patient’s wealth status. This is the case for insulin and some oral antidiabetic drugs, treatment services for child malnutrition or management of pregnancy-related conditions, among other services.

With regard to household head’s occupation, our results seem suggesting a differential health-seeking behaviour between households headed by petty trader or farmer, and those headed by formal salaried. Farmers and petty traders are likely to have a lower socio-economic position and education level, and more limited access to specialized and often expensive health services offered at hospitals compared to formal salaried [50]. On the other hand, the quality of services offered at primary healthcare level may be deemed sub-optimal by richer and more educated individuals. This finding supports the claim that one of the most efficient ways of bringing quality care to the most vulnerable in poor and post-conflict fragile contexts is to invest in strong primary healthcare schemes [51,52].

### Study limitations

This is one of the first studies to apply a comprehensive approach to measuring healthcare needs and its association with health facility utilisation at the community level in post-conflict settings. Using a sample of people with self-reported diabetes, hypertension and mothers of infant with malnutrition selected from villages around the health centres limits the generalisability of our findings. Besides, this study may not reflect the situation of the most hard-to-reach and remote areas with active armed conflict in South Kivu and where the health needs and service utilisation may be different. Therefore, additional investigations are needed to cover such contexts. That our sample size was not estimated for this study is less likely to introduce a bias given the high precision of point intervals reflected in narrow confidence intervals. We did not do post-hoc power calculations since this practice is increasingly seen as obsolete and discouraged [53]. Although we used a cross-sectional study design, the strength of the associations observed between explanatory factors and HFU despite multivariable adjustment for extensive confounders suggests the associations are real. Our findings may still be subject to residual confounding and unmeasured factors such as distance to health facilities or financial inaccessibility [54]. Furthermore, some of the study participants who reported illness might have not necessarily felt the need to attend a health facility, therefore caution needs to be exerted while interpreting findings about the level of HFU. Finally, the effect estimates might have been influenced by both social desirability bias and the interviewer effect as data collectors were nurses living in the study sites and the questionnaire could not be self-administered given the low literacy level in these predominantly rural settings. This may have led participants to conceal information about seeking care from informal sources, in which case modern HFU will end up over reported.

## Conclusion

In this post-conflict setting, healthcare need was significantly associated with health facility utilisation whereby those with greater reported needs were more likely to utilise health facilities. Primary healthcare facilities were the first resort for a vast majority of respondents. There is a need to rethink priorities regarding the provision of quality  primary healthcare in post-conflict settings. This is critical to achieving the UHC goal of advocating quality health for all.

## Paper context

The disruptive effect of socio-political instability and conflict on the health systems is likely to exacerbate inequities in health in fragile settings. We examined the association between healthcare needs and health facility utilisation in eastern DR Congo. Our study suggests that vulnerable people are using primary health facilities as a first resort. Improving the availability and quality of primary healthcare is critical to the achievment of universal health coverage in post-conflict settings.
